# Heterogeneous estimates of influenza virus types A and B in the elderly: Results of a meta‐regression analysis

**DOI:** 10.1111/irv.12550

**Published:** 2018-03-23

**Authors:** Donatella Panatto, Alessio Signori, Piero L. Lai, Roberto Gasparini, Daniela Amicizia

**Affiliations:** ^1^ Department of Health Sciences University of Genoa Genoa Italy; ^2^ Interuniversity Research Center on Influenza and other Transmissible Infections (CIRI‐IT) Genoa Italy

**Keywords:** elderly, influenza types A and B, meta‐regression analysis

## Abstract

Influenza has many age‐dependent characteristics. A previous systematic review of randomized controlled trials showed that the detection rate of influenza B was higher in children than in non‐elderly adults. However, no comprehensive reviews have targeted the elderly, who carry the main burden of disease. We aimed to quantify the relative detection rates of virus types A and B among the elderly, to identify factors affecting these proportions, and to compare type distribution among seniors and younger age‐classes. A comprehensive literature search was conducted to identify multiseason studies reporting A and B virus type distributions in the elderly. A random‐effects meta‐analysis was planned to quantify the prevalence of type B among elderly subjects with laboratory‐confirmed influenza. Meta‐regression was then applied to explain the sources of heterogeneity. Across 27 estimates identified, the type B detection rate among seniors varied from 5% to 37%. Meta‐analysis was not feasible owing to high heterogeneity (*I* ^2 ^= 98.5%). Meta‐regression analysis showed that study characteristics, such as number of seasons included, hemisphere, and setting, could have contributed to the heterogeneity observed. The final adjusted model showed that studies that included both outpatients and inpatients reported a significantly (*P *=* *.024) lower proportion than those involving outpatients only. The detection rate of type B among the elderly was generally lower than in children/adolescents, but not non‐elderly adults. Influenza virus type B has a relatively low detection rate in older adults, especially in settings covering both inpatients and outpatients. Public health implications are discussed.

## 1 INTRODUCTION

Like that of many other infectious diseases, the epidemiology of influenza displays several age‐dependent features. First of all, influenza attack rates are generally higher in children than in adults and, especially, the elderly. For instance, a meta‐regression analysis by Jayasundara et al^1^ has shown that the natural attack rate is about four times higher (15.2% vs 3.5%) in children than in adults. Our previous research^2^ documented that, in ten consecutive seasons, children aged 0‐14 years had the highest cumulative incidence of influenza‐like illness (ILI), followed by 15‐ to 64‐year‐olds, while the lowest cumulative incidence was constantly reported in the elderly. On the other hand, despite the lower incidence rates, influenza‐attributable hospitalizations and mortality are highest among the elderly; on average, about 90% of influenza‐related deaths occur in people aged 65 years or older.^3^, ^4^, ^5^

The magnitude of incidence is not the only age‐related attribute of epidemic curves; the relative timing of the onset and peak of an epidemic is also likely to differ among age‐groups. Although it is as yet unclear who exactly “drives” epidemics (eg, both pre‐school and school children^6^, ^7^ and high‐school students^8^ have been implicated), it seems that younger populations are more likely to spread the virus in their households. However, the relative timing of seasonal epidemics also depends on the circulating (sub)type.^9^

Available virological data^1^ also support the above‐mentioned thesis concerning the age dependency of influenza, in that different influenza (sub)types affect different age‐classes in different ways. Let us remember that there are three “classic” influenza virus types: A, B, and C. Of these, influenza virus types A (IVA) and B (IVB) are of major public health interest. On the basis of two major surface glycoproteins, namely hemagglutinin (H) and neuraminidase (N), IVA is further divided into subtypes; A (H1N1) and A (H3N2) have been clearly dominant for several years. IVB, by contrast, has evolved into two distinct lineages, Victoria and Yamagata.^10^, ^11^, ^12^ Moreover, a fourth type of influenza virus, dubbed “D”, has recently been proposed, whose role in humans is uncertain.^13^, ^14^ The meta‐regression analysis cited above^1^ highlighted the fact that IVB has a relatively greater impact on children than on adults, the ratio IVB/IVA being 0.45 in children and 0.25 in adults. These estimates are of importance to public health planning and policies. For instance, owing to the lack of data on vaccine efficacy/effectiveness (VE) in the various age‐classes, the relative advantage of the recently introduced quadrivalent influenza vaccine (QIV) (which contains both IVB lineages) over trivalent (TIV) formulations has usually been calculated mathematically^15^, ^16^, ^17^ from a set of epidemiological parameters. These have included, for example, the mean relative detection rate (DR) of IVB, the mean level of lineage mismatch between the IVB included in TIV and that in circulation, and a meta‐analytically obtained level of cross‐lineage protection provided by TIV. It is, however, evident that, while the DR of IVB in children is about twice as high as that seen in adults, the relative advantage of QIV over TIV would be significantly greater among younger populations. Moreover, a somewhat age‐dependent cost‐effectiveness profile of QIV has recently been demonstrated in the United Kingdom (UK):^18^ QIV would be cost‐effective in children with an increased cost of up to £6.36 per dose; if, however, the program was extended to at‐risk individuals aged <65 years and further to all elderly subjects, the maximum incremental cost per dose would be £1.84 and £0.20, respectively.

This study had three objectives, two of which were co‐primary and one secondary. The co‐primary objectives were (1a) to quantify the proportions of IVA and IVB in relation to the total number of viruses detected (IVA+IVB) among the elderly, and (1b) to identify factors influencing the relative prevalence of influenza virus types. The secondary objective was to compare the distribution of IVA and IVB among the elderly with that observed among younger age‐classes.

Considering the previous findings,^1^ we hypothesized an uneven distribution of IVA and IVB among different age‐classes, whereby the impact of IVB was relatively greater in younger people. This study is of importance for all relevant stakeholders for at least two reasons. First, the previously published meta‐regression^1^ considered only randomized controlled trials (RCTs) and therefore did not include nationally or regionally representative influenza surveillance data. Although RCTs allow both prospective monitoring of influenza attack rates and more rigorous bias control, they are usually conducted with the aim of comparing two or more different strategies, in a limited time‐frame and with a limited number of participants; thus, they may not fully reflect the “real‐world” scenario. Population‐based surveillance studies may at least partially address these issues.^19^ Moreover, the analysis by Jayasundara et al^1^ was not able to establish IVA/IVB attack rates among the elderly, owing to the paucity of studies and missing information. Other recently published reviews on the epidemiology and burden of IVB have considered, for instance, single countries^20^ or have not aimed to analyze IVA/IVB distribution patterns from the perspective of age.^21^ Second, no comprehensive reviews on the impact of IVA/IVB in the elderly are available; elderly people are the primary target of annual influenza vaccination in all industrialized and many developing countries. Given the variety of influenza vaccines available for immunization (such as trivalent and quadrivalent, adjuvanted and non‐adjuvanted),^22^ insights from this epidemiological review could be helpful in future pharmacoeconomic and health technology assessment (HTA) evaluations aimed at establishing an equitable vaccination policy.

## 2 METHODS

### 2.1 Compliance with international standards

We followed the “Methodological guidance for systematic reviews of observational epidemiological studies reporting prevalence and cumulative incidence data” proposed by researchers from the Joanna Briggs Institute.^23^ The meta‐analyses of observational studies in epidemiology (MOOSE) checklist^24^ were also consulted.

### 2.2 Study endpoints, population, and key definitions

In accordance with the above‐described study objectives, the study endpoints were as follows:

1a Proportion of IVB to the total number of viruses detected (IVA+IVB) in the elderly;
1b Factors associated (see below) with the relative frequency of IVB in the elderly;
2 Relative risk (RR) of detecting IVB in the elderly as compared with younger age‐classes.

We defined “elderly subjects” according to the two most widely used cutoffs of ≥60 and ≥65 years.^25^

Laboratory‐confirmed influenza cases were defined as cases that tested positive in diagnostic assays described by Vemula et al^26^ including mainly viral cultures and reverse transcriptase polymerase chain reaction (PCR).

Given both the different timing of influenza epidemics in the Southern and Northern Hemispheres and the fact that several studies may not have reported the exact surveillance period, the definition of an influenza season took into account the study location, and was adapted from Caini et al.^27^ For example, the study period of “2012‐14” refers to the three consecutive seasons (2012‐13, 2013‐14, and 2014‐15) in the Northern Hemisphere or to the three consecutive years (2012, 2013, and 2014) in the Southern Hemisphere.

### 2.3 Search strategy

A comprehensive literature search was conducted; this included both automatic and manual modalities. As the authors of this study were aware that several studies^27^, ^28^, ^29^, ^30^, ^31^ on the outcomes of interest were available, the automatic search strategy was constructed in several steps until all known studies appeared among the search results. To do this, it was first assumed that studies reporting the frequency of IVB would also report that of IVA. Even if this were not the case, the frequency of IVA could have easily been imputed by subtracting IBV isolates from the total number of isolates. Studies that quantified IVB were sought using a combination of both MeSH (medical subject headings) terms and text words. The search strings regarding the study population, that is the elderly, were adapted from Jefferson et al^32^ The algorithm was developed by two investigators (AS and DP) and approved by the whole team. The search was limited to the period of “1990‐Current.” No other filters were applied. The search strategy was first implemented in Medline via Ovid (Box S1) and then adapted to Embase. The search was performed on June 26, 2017.

Subsequently, we automatically searched the so‐called gray literature; this search was conducted at greylit.org. Given the limited number of available records, the only search term used was “influenza.”

The automatic search was completed by the manual search; this was done by means of the standard citation‐tracking method of the studies included.

### 2.4 Study selection process

After the removal of duplicates, the results of each automatic search were pooled into a single spreadsheet, and a first screening was performed to eliminate clearly irrelevant titles. The remaining set of papers underwent abstract screening. In these first two steps, articles were removed if they: (i) did not refer to laboratory‐confirmed influenza or (ii) had a clearly different study population (ie, <60 years). Subsequently, full texts of potentially eligible papers were assessed; to be included in the review, these had to meet all of the following inclusion criteria:

1. Separate data on IVA and IVB among people aged ≥60/65;
2. Coverage of at least five consecutive influenza seasons;
3. Total number of infections detected (IVA+IVB) among the elderly >100.

Seroepidemiological surveys were excluded.^33^

During the selection process, we realized that some studies had been conducted by the same research groups, and that the same databases had been used to answer different research questions. In such cases, we selected the study with the highest number of viruses detected and/or covering the most seasons.

### 2.5 Data extraction and abstraction

The data were extracted and inserted into a spreadsheet by DP; this procedure was then cross‐checked by DA. The following parameters were extracted: first author, year of publication, country/location, study setting/surveillance system, study period, virus detection and characterization laboratory methods, age‐classes for which data of interest were reported, total, and by‐type age‐class‐specific absolute number of influenza viruses detected.

When data were not readily available for extraction, the authors of papers (i) reporting results only through percentages/charts, but without a clearly stated denominator or (ii) indicating an age stratification in the “Methods” but not reporting age‐specific data in the “Results” were contacted by email for further details.

Any instances of mixed infections (IVA + IVB) or type C infections were excluded from the total count. Although some were detected, these, however, accounted for a negligible proportion.

Some studies reported relative frequencies of IVA and IVB on a scale that was different (eg, % distribution of IVB among different age‐classes) from the study endpoints (ie, % distribution of IVA and IVB in a given age‐class). However, as these studies also reported the total number of IVA and IVB, the absolute numbers of interest in a given age‐class was easily recalculated.

Age categorization was performed once the study selection process had been deemed completed. In a few studies that further broke down the elderly population into categories, these estimates were summed to form a single age category of ≥60/65 years. The main challenge lay in classifying the pediatric population; indeed, most studies reported data of interest separately for age‐groups of 0‐4/5 and 4/5‐14/17/18 years, while some combined the two latter groups into a single category. We therefore used the following classification: “young children” (0‐4/5 years), “older children/adolescents” (4/5‐14/17/18 years), “children/adolescents” (0‐14/17/18 years), “adults” (14/17/18/25‐60/64 years), and “elderly” (≥60/65 years).

### 2.6 Study variables

The following variables were considered in the analysis:

1. Number of influenza seasons;
2. A dichotomous variable indicating the hemisphere;
3. Absolute centroid latitude of study location;
4. A dichotomous variable indicating whether the 2009 pandemic fell within the study period;
5. A categorical variable with three levels indicating the study setting [outpatients only, inpatients/severe acute respiratory infection (SARI) only, both outpatients and inpatients/SARI].

### 2.7 Quality assessment

Following consultation of the World Health Organization's (WHO) manual “Global Epidemiological Surveillance Standard for Influenza,”^34^ we realized that some of our endpoints and inclusion criteria could be regarded as quality attributes. Indeed, we considered only laboratory‐confirmed influenza, studies with clear virus type and age‐class distributions, a sufficient period of time [to avoid short‐period studies driven by a single (sub)type] and number of viruses detected among the study population. This could be described as the “minimum criteria for inclusion” approach.^23^ A formal critical appraisal was, however, conducted by means of a 9‐item tool created by researchers from the Joanna Briggs Institute.^23^

However, a difficulty emerged regarding the representativeness of the study population (item 1 of the appraisal tool). Indeed, representativeness is a multifaceted issue. It may be sentinel or non‐sentinel, inpatient or outpatient, cover a geographically representative area or not, etc.^35^ The distribution of swabs taken from patients of different ages often does not reflect the age‐structure of the general population; indeed, the probability of being tested for influenza differs significantly among single age‐classes.^36^ Regarding the surveillance system, it was deemed that the estimates included were representative within the context of their setting and spatial coverage. The study setting was, however, considered a priori to be a potential confounder (see above). By contrast, with regard to age‐class‐specific representativeness, we realized that this was only partly addressed by including studies with at least 100 positive tests among the elderly. We therefore compared the proportion of positive tests from the elderly with the percent of the elderly population, as reported by the World Bank (mean annual proportion of the study period).^37^ The age‐adjusted proportion of positive tests from the elderly was then used in meta‐regression as a potential predictor of the study's primary endpoint.

### 2.8 Statistical analysis

Spearman's ρ coefficient was used to measure correlation between the number of viruses detected from the elderly and the weight of the elderly in the general population, as per World Bank.^37^ Subsequently, a simple linear model was constructed in order to adjust the proportion of positive tests from the elderly to the percent of the elderly population in a given country.

Given that we expected a high level of heterogeneity among single studies,^1^ and in line with explicit recommendations from Munn et al^23^ on the pooling of prevalence estimates, all models were planned a priori to be random‐effects. Heterogeneity was quantified by both *I* ^2^ and *Q* test. In particular, we expected to pool the proportion of IVB with the total number of viruses detected (IVA+IVB) in the elderly (endpoint 1a) and express this outcome as a raw proportion (by applying the arcsine square‐root transformation). However, no pooled result was retained in the case of *I* ^2 ^> 85%.^38^

We decided a priori to carry out two types of sensitivity analysis. The first would involve the sequential omission of estimates from single studies. In the second, we would omit studies that could potentially include overlapping participants, that is, studies conducted in the same territory and/or covering overlapping influenza seasons. Thus, only studies with the largest number of estimates from a given study location would be retained. Publication bias was planned to be checked by means of both the visual inspection of funnel plots and Egger's test.

To explain the heterogeneity observed and/or find factors associated with endpoint 1a, a set of univariable meta‐regressions (endpoint 1b) was carried out; the potential predictors were described earlier in the text. Independent variables deemed significant (*P *<* *.05) on univariable analysis were then included in a multivariable model, in which all *P* ‐values were adjusted for multiple testing.

The RR was used as a measure for our secondary endpoint, that is, to compare the IVB DR between the elderly and younger age‐classes.

All analyses were conducted in Stata version 14 (StataCorp LP, College Station, TX, USA) and MetaXL version 5.1 (Epigear International).

## 3 RESULTS

### 3.1 Characteristics of studies included

The automatic search produced a total of 6733 unique records. Following duplicate removal (N* *=* *946) and the screening of titles and abstracts (N =* *5787), a total of 43 records were deemed to be worthy of further evaluation and the corresponding full texts were assessed. Of these, 16 papers^27^, ^28^, ^29^, ^30^, ^31^, ^39^, ^40^, ^41^, ^42^, ^43^, ^44^, ^45^, ^46^, ^47^, ^48^, ^49^ met all inclusion criteria and were included. In the study by Caini et al^27^ only 12 of 26 study locations had a sufficient number (≥100) of isolates among the elderly. Moreover, we identified three potentially eligible papers by Mosnier et al^31^, ^43^, ^50^ that used the same database and almost the same time‐frame. However, the number of reported isolates varied. Specifically, one paper^31^ dealt exclusively with the elderly, while other two^43^, ^50^ also reported data of interest regarding younger age‐classes. We therefore proceeded in the following way. As the first paper^31^ reported the largest number of viruses detected among the elderly, this study was included to address endpoints 1a and 1b. For endpoint 2, however, we selected one of the other two studies,^43^ as it presented a higher number of positive tests in the whole population. In any case, the relative estimates reported in these three studies were close to one another. The manual search did not produce results. An additional source of data^51^ was suggested by a peer‐reviewer. In sum, a total of 17 papers corresponding to 27 IVB prevalence estimates were included in both the qualitative and quantitative assessments. The whole process of data selection is depicted in Figure S1.

The pre‐specified characteristics of the studies included are reported in Table 1. Most (23/27) prevalence estimates were from the Northern Hemisphere and covered 19 different countries. All included studies were published/extracted within a relatively short period of time (2013‐2017). The median number of seasons covered was 8 [interquartile range (IQR): 6‐12] and most (22/27) prevalence estimates included the 2009 pandemic. The setting of most (15/27) estimates was from both outpatient and inpatient/SARI surveillance. The median number of IVA+IVB among the elderly was 737 (IQR: 198‐2303).

**Table 1:** Characteristics of studies included

First author	Year of publication	Study location	|Latitude| (Hemisphere)	Period	Setting/surveillance	Methods of detection and/or characterization	Age‐classes (y) extracted	Ref
Chan	2013	Hong Kong	22.3 (N)	2000‐10^a^	Inpatients	IFA, culture, PCR	0‐4, 5‐9, 10‐14, 15‐64, 65‐79, >79	39
Nguyen	2013	Vietnam	16.2 (N)	2006‐10	Outpatients (ILI)	PCR	0‐14, 15‐24, 25‐64, >64	40
Heikkinen	2014	Finland	64.0 (N)	1999‐12^a^	Statistical Database of the Infectious Diseases Register	Sequencing	0‐4, 5‐9, 10‐14, 15‐19, 20‐29, 30‐39, 40‐49, 50‐59, 60‐69, ≥70	41
Caini	2015	Australia	25.0 (S)	2001‐12	Outpatients, SARI	PCR, serology, culture, HI, sequencing	0‐4, 5‐17, 18‐64, ≥65	27
Caini	2015	Chile	30.0 (S)	2008‐12	Outpatients, inpatients, SARI	PCR, culture, HI, sequencing	0‐4, 5‐17, 18‐64, ≥65	27
Caini	2015	China (North)	39.9 (N)	2005‐12	Outpatients	PCR, culture, HI	0‐4, 5‐17, 18‐64, ≥65	27
Caini	2015	China (South)	31.2 (N)	2006‐12	Outpatients	PCR, culture, HI	0‐4, 5‐17, 18‐64, ≥65	27
Caini	2015	El Salvador	13.8 (N)	2006‐13	Outpatients, SARI	PCR, culture, IFA, WHO referencing	0‐4, 5‐17, 18‐64, ≥65	27
Caini	2015	England	51.5 (N)	2003‐13	Outpatients, inpatients	PCR, culture, HI, sequencing	0‐4, 5‐17, 18‐64, ≥65	27
Caini	2015	Guatemala	15.5 (N)	2006‐13	Outpatients, SARI	PCR, culture, IFA, WHO referencing	0‐4, 5‐17, 18‐64, ≥65	27
Caini	2015	Italy	43.0 (N)	2002‐12	Outpatients, inpatients, SARI	PCR, HI, sequencing	0‐4, 5‐17, 18‐64, ≥65	27
Caini	2015	New Zealand	42.0 (S)	2000‐12	Outpatients, inpatients, SARI	Sequencing, WHO referencing	0‐4, 5‐17, 18‐64, ≥65	27
Caini	2015	Nicaragua	13.1 (N)	2007‐13	Outpatients, SARI	PCR, culture, IFA, WHO referencing	0‐4, 5‐17, 18‐64, ≥65	27
Caini	2015	Singapore	1.3 (N)	2007‐12	Outpatients	PCR, HI, sequencing	0‐4, 5‐17, 18‐64, ≥65	27
Caini	2015	Vietnam	16.2 (N)	2006‐13	Outpatients, SARI, other	PCR, culture, HI, sequencing	0‐4, 5‐17, 18‐64, ≥65	27
Hinds	2015	Canada (Manitoba)	55.0 (N)	1993‐08	Outpatients, inpatients	Culture, PCR, rarely other	0‐1, 2‐4, 5‐9, 10‐14, 15‐24, 25‐44, 45‐64, 65‐74, ≥75	42
Mosnier^b^	2015	France	47.0 (N)	2003‐13	Outpatients (ARI)	EIAs, culture, HI, PCR	0‐4, 5‐14, 15‐64, ≥65	43
Wang	2015	Hong Kong	22.3 (N)	2004‐10	Inpatients	IFA, culture, PCR	0‐5, 6‐17, 18‐39, 40‐64, ≥65	44
Yang	2015	Hong Kong	22.3 (N)	2004‐13	Outpatients, inpatients	IFA, culture, PCR	0‐4, 5‐17, 18‐64, ≥65	45
Zhao	2015	China (Shanghai)	31.2 (N)	2009‐14	Outpatients (ILI)	PCR, culture, HI, sequencing	0‐2, 2‐5, 6‐17, 18‐64, ≥65	46
Kandeel	2016	Egypt	26.0 (N)	2007‐14	Inpatients (SARI)	PCR, WHO referencing	0‐4, 5‐17, 18‐64, ≥65^c^	47
Qi	2016	China (Chongqing)	46.5 (N)	2011‐15	Outpatients and/or ED visits (ILI)	PCR	0‐4, 5‐14, 15‐24, 25‐59, ≥60	48
An der Heiden	2017	Germany	51.0 (N)	2001‐15	Medically attended ARI	PCR	0‐4, 5‐14, 15‐34, 35‐59, ≥60	28
Chiarella	2017	Spain (Madrid)	40.4 (N)	2010‐16	Outpatients, inpatients	Rapid tests confirmed by PCR	0‐4, 5‐18, 18‐30, 31‐45, 46‐65, >65	29
Coleman	2017	Canada (Toronto)	43.7 (N)	2004‐14	ILI requiring hospitalization	Culture, IFA, EIA, PCR	<15, 15‐64, ≥65	49
Moa	2017	Australia	25.0 (S)	2001‐14	Outpatients, SARI	PCR, serology, culture, HI, sequencing	0‐4, 5‐9, 10‐19, 20‐49, 50‐64, 65‐84, ≥85	30
Mosnier^d^	2017	France	47.0 (N)	2003‐14^a^	Outpatients (ARI)	EIAs, culture, HI, RT‐PCR	≥65, 65‐69, 70‐74, ≥65	31
CDC	2017	United States	40.0 (N)	1997‐17	Outpatients, inpatients, SARI	PCR, culture, HI, sequencing	0‐4, 5‐24, 25‐64, ≥65	51

a Pandemic period was excluded.

b Study used for the secondary endpoint.

c Owing to the presence of raw data, age was categorized as majority of studies.

d Study used for the primary endpoint.

All studies were judged to be of good quality in all domains, except for checklist item 1 regarding representativeness. Indeed, on median, only 6.2% (IQR: 3.3%‐13.8%) of infections detected were from the elderly population, and the correlation between the number of positive tests from the elderly and the weight of the elderly in the general population was low (Spearman's ρ = 0.45), although statistically significant (*P *=* *.020). Age adjustment of the proportion of IVA + IVB from the elderly enabled us to significantly increase the representativeness of the data.

### 3.2 Detection rate of influenza type B in the elderly and its possible determinants

**Figure 1:**
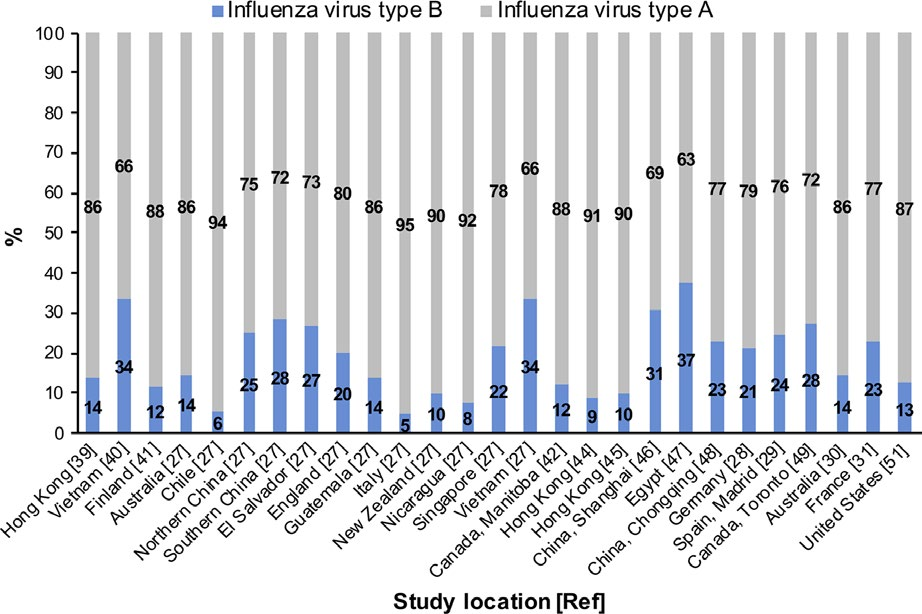
Detection rates of influenza types A and B among the elderly, by study

There was a 7‐fold difference in estimates of IVB prevalence among the elderly, which ranged from 5.1% to 37.4% (Figure 1). The level of heterogeneity was very high (*I* ^2 ^= 98.5%; *Q *=* *1752.7, *P *<* *.001), which did not allow us to obtain a pooled estimate.

To highlight possible sources of the heterogeneity observed, a set of univariable meta‐regressions was performed (Table 2). Three predictors were found to be statistically associated with the relative DR of IVB among seniors. First, the IVB DR was significantly higher (*P *=* *.045) in the Northern Hemisphere than in the Southern. Second, studies that involved only outpatients were significantly (*P *<* *.001) more likely to report higher IVB DRs than those comprising both in‐ and outpatient surveillance systems. Third, studies covering a wider time‐frame were more likely (*P *=* *.028) to report lower IVB DRs. These single models explained 11% to 32% of variance. As per peer‐reviewer suggestion, we performed a post hoc meta‐regression with the predictor of unadjusted (to the population structure) percent of IVB among the elderly in relation to the number of viruses detected in the whole population; no statistically significant (*P *=* *.25) association emerged.

**Table 2:** Univariable meta‐regression models to predict influenza type B detection rate among the elderly

Variable	Level	Estimate (95% CI)	*P*	*R* ^2^, %
Latitude	–	−0.0008 (−0.0032, 0.0016)	.50	0
Hemisphere	Northern	Ref	–	10.7
	Southern	−0.0919 (−0.1817, −0.0021)	.045	
Setting	Outpatient only	Ref	–	31.9
	Inpatient only	−0.0457 (−0.1370, 0.0456)	.33	
	Inpatient and outpatient/SARI	−0.1147 (−0.1806, −0.0488)	<.001	
Pandemic period included	No	Ref	–	0
	Yes	0.0277 (−0.0617, 0.1171)	.54	
Number of seasons	–	−0.094 (−0.0178, −0.0010)	.028	12.6
Weighted % isolates among the elderly	–	−0.0069 (−0.0150, 0.0011)	.088	6.4

In the multivariable model, which included only significant variables determined at the previous step, the only statistically significant predictor was “Setting” (*P *=* *.024 for “outpatient only” vs “in‐ and outpatient” surveillance systems; *P *=* *.42 “inpatient only” vs “outpatient only” surveillance systems). By contrast, the variables “Hemisphere” (*P *=* *.32) and “Number of seasons” (*P *=* *.25) did not prove to be significantly associated with the outcome.

### 3.3 Detection rate of influenza type B in the elderly: comparison with younger age‐classes

Table 3 compares IVB DRs among the elderly and younger age‐classes in terms of RRs (elderly vs younger populations). In 80% (20/25) of studies, IVB DRs were lower among the elderly than among younger children. Direct comparison of elderly vs older children/adolescents revealed that 92% (22/24) of studies had a RR<1; the effect sizes were generally higher than in the previous comparison “elderly vs younger children.” A similar pattern was observed when the two above‐mentioned pediatric age‐classes were combined together. By contrast, the head‐to‐head comparison of elderly and non‐elderly adults produced somewhat controversial findings: Higher and lower IVB DRs in the elderly were almost evenly distributed among single studies.

**Table 3:** Relative risk of influenza type B detection among the elderly in comparison with younger age‐classes

Location [Ref]	Elderly vs children	Elderly vs adolescents	Elderly vs children/adolescents	Elderly vs adults
Hong Kong^39^	0.65	0.33	0.48	0.67
Vietnam^40^	NA	NA	0.94	1.18
Finland^41^	0.70	0.31	0.44	0.42
Australia^27^	0.91	0.66	0.73	1.04
Chile^27^	0.71	0.31	0.47	0.94
China (North)^27^	0.66	0.77	0.73	0.95
China (South)^27^	0.89	0.93	0.92	1.05
El Salvador^27^	0.89	1.51	1.16	1.79
England^27^	0.95	0.66	0.71	0.87
Guatemala^27^	0.91	0.88	0.90	2.27
Italy^27^	0.81	0.27	0.37	0.83
New Zealand^27^	0.57	0.36	0.43	0.70
Nicaragua^27^	0.42	0.39	0.40	2.92
Singapore^27^	0.96	0.64	0.66	0.96
Vietnam^27^	1.00	0.82	0.91	1.16
Canada (Manitoba)^42^	0.51	0.26	0.39	0.43
France^43^	1.29	0.90	1.03	1.35
Hong Kong^44^	0.58	0.45	0.52	1.05
Hong Kong^45^	0.80	0.48	0.61	1.01
China (Shanghai)^46^	0.91	0.77	0.81	0.98
Egypt^47^	1.29	1.05	1.14	1.28
China (Chongqing)^48^	0.74	0.48	0.55	1.17
Germany^28^	1.17	0.59	0.71	0.94
Spain (Madrid)^29^	1.11	0.49	0.83	1.25
Canada (Toronto)^49^	NA	NA	0.96	1.38
Australia^30^	0.93	0.59	0.68	0.95
United States^51^	0.86	NA	0.67^a^	0.91

a The age category is 5‐24 y; it therefore also includes young adults.

None of the above‐reported pairwise comparisons were poolable owing to the very high heterogeneity (*I* ^2 ^> 94%; *Q *>* *198, *P *<* *.001).

## 4 DISCUSSION

The present study established the average multiseason proportions of IVA and IVB among the elderly in different settings and geographical areas; IVA was by far prevalent, while IVB accounted for less than a quarter of cases in most settings. We then demonstrated that the type‐specific DRs among the elderly may depend on study characteristics, such as the type of surveillance system. Finally, the DR of IVB among seniors tended to be substantially lower than in children and adolescents, but not non‐elderly adults.

To the best of our knowledge, our study provides the first comprehensive review of the IVB proportion among the elderly. It may therefore be seen as a valuable addition to the previously cited meta‐regression study of RCTs on the natural attack rate of IVA and IVB.^1^ Indeed, that study did not quantify the natural attack rate of IVA/IVB in the elderly, as two of the only three studies identified did not report data of interest, while the third reported a natural attack rate of zero. The paucity of studies identified is not surprising; indeed, randomized placebo‐controlled trials have been very uncommon (at least in recent years) in this population group for purely ethical reasons, in that annual influenza vaccination is recommended for the elderly.^52^ This is why epidemiological and surveillance evidence is of importance.^53^

However, a substantial difference in IVA/IVB DRs, which determined a very high heterogeneity, was reported by single studies. This fact did not allow us to retain any pooled estimate. The meta‐regression approach helped us to identify possible confounders that may have been responsible for the heterogeneity observed in DR estimates. In the final adjusted model, we ascertained that the study setting/surveillance system may have contributed significantly to the heterogeneity observed. Specifically, the regression coefficients for “outpatients only” were lower than those for “inpatients only” and “in‐ and outpatients”, although only the latter reached an α < .05. The observed non‐significance between “outpatients only” and “inpatients only” could be attributed to the relatively low number of studies involving the latter category.

The observed gap between study settings is probably attributable to several factors. Although recent research has suggested that several clinical features and outcomes of IVA and IVB are similar,^54^, ^55^ it is largely unknown the relative frequency of hospitalization in IVA and IVB patients of different ages.^56^ Indeed, the frequency of hospitalization due to IVA/IVB may not reflect the epidemiological picture of circulating (sub)types. For instance, in the United States during the season 2007/2008, IVB was detected more frequently at population level, while the number of IVB hospitalized patients was about the half of IVA hospitalized patients.^54^ Other factors that may explain the difference observed are representativeness and data quality. The outpatient ILI surveillance system may be more nationally representative, while inpatient/SARI surveillance may provide more in‐depth and higher‐quality data.^57^ As it has been suggested^57^, ^58^ that both surveillance types are of merit and should be regarded as complementary, we believe that the estimates of IVA/IVB DRs from studies including both out‐ and inpatients better reflect reality.

Results regarding our secondary outcome confirmed the widely held thesis^59^ that IVB mainly affects young populations. Indeed, we found that young children and older children/adolescents usually had a higher probability of IVB detection than the elderly, while no evident pattern could be observed between elderly and non‐elderly adults. These results may be explained by the gradual exposure to IVB during the first two decades of life;^60^ indeed, virtually all elderly subjects have been exposed to IVB during their lives.^61^ However, the question of cross‐lineage IVB protection remains controversial: some studies have found considerable cross‐protection,^62^, ^63^ while others^64^, ^65^ have reported limited or no cross‐protection. The most recent, and one of the most comprehensive, meta‐studies was conducted by Beyer et al^61^ and may shed light on this issue. These authors found that mismatch of the IVB lineage (that included in TIV) impacted heavily on VE in young individuals (31.8%‐73.3%) but not in the elderly (2.4%‐3.4%). However, why individuals aged 4/5‐14/17/18 (and not 0‐4/5) years are major IVB spreaders remains largely unknown; future research into this is warranted.

Our results may have public health policy implications. For instance, in the elderly, the age‐class in which traditional influenza vaccines are often poorly immunogenic owing to immunosenescence^32^, ^66^ and IVB causes a relatively low burden, the implementation of enhanced vaccine formulations, such as MF59‐adjuvanted TIV or high‐dose TIV, may have significant advantages. Indeed, these vaccines have been shown to be more immunogenic and effective in the senior population.^38^, ^67^, ^68^, ^69^, ^70^, ^71^ By contrast, QIVs may offer a substantial benefit in children and adolescents (especially those aged 5‐17 years) owing to the significant presence of IVB in this age‐class and the relatively high level of mismatch between the dominantly circulating IVB lineage and that included in TIVs. From our personal experience, we have learned that all available influenza vaccines differ in several aspects, and that each one is more appropriate for a given population group, a notion that is also exemplified by a recent paper by Bonanni et al^72^ on the appropriateness of different influenza vaccines available in Italy. Indeed, some Italian regions have been implementing “age/risk‐group” vaccine differentiation.^73^ This differentiation could also be attractive from the economic point of view; indeed, it has been estimated^74^ that a strategy in which MF59‐adjuvanted TIV is used exclusively in the elderly, while QIV is used in younger age‐groups, is the most cost‐effective from the payer perspective.

This review is not without limitations. First, although we performed a comprehensive literature search, we acknowledge that some relevant data might not have been found or not have been publicly available. Ideally, our search strategy should have involved common search engines, to locate appropriate national, regional or local information sources that are not indexed in the normally used scientific databases. However, the low specificity of the common search engines and the impossibility of constructing a comprehensive search tree (which would involve hundreds of ad hoc queries in different languages) did not allow us to proceed in this way. We tried to limit this shortcoming by searching the “gray literature”, although this proved fruitless. In any case, we believe that both age‐ and (sub)type‐specific data should be routinely reported by the surveillance systems.

Second, our results come from a global perspective and should be regarded as average multiseason estimates. It is evident that, during seasons with an overall predominance of IVB, the DRs of IVB will be higher among the elderly. However, it has been demonstrated (data from 12 European countries)^75^ that in the 2012/2013 season, when IVB was clearly dominant, the elderly had a significantly lower risk of IVB detection than 5‐ to 14‐year‐olds (RR 0.67, *P *<* *.001).

Third, the meta‐analytic estimates were subject to very high heterogeneity and were therefore not retained. It should, however, be pointed out that, from our personal experience, meta‐analytic prevalence estimates are very often associated with very high heterogeneity. Indeed, in the meta‐analysis conducted by Jayasundara et al^1^, ^2^ values were constantly >90%. There are several ways of dealing with heterogeneity, including: (i) choosing a fixed‐effects or a random‐effects model; (ii) changing the statistical metric; (iii) excluding studies; (iv) omitting meta‐analyses and conducting subgroup analysis or meta‐regression.^76^ Here, we chose a more conservative approach; we omitted pooled results and carried out a set of meta‐regressions to explain the heterogeneity observed. If, on the other hand, we had reported the pooled estimates relative to our endpoint 1a (IVB DR among the elderly), these would have been 14.0% (95% CI: 13.8%‐14.1%) and 17.8% (95% CI: 15.8%‐19.8%) in the fixed‐ and random‐effects models, respectively (results not shown).

## CONFLICT OF INTEREST

The authors have no conflict of interest.

## STATEMENT

To our knowledge, this is the first paper to review the relative contribution of influenza virus types A and B in the elderly from the global perspective. We also show that the virus type distribution may depend on the study setting.
