# Supplementary figures and images for: Heterogeneous estimates of influenza virus types A and B in the elderly: Results of a meta‐regression analysis
Source: Influenza Other Respir Viruses. 2018 Mar 23;12(4):533–43. doi: 10.1111/irv.12550 (PMC6005586; doi:10.1111/irv.12550)

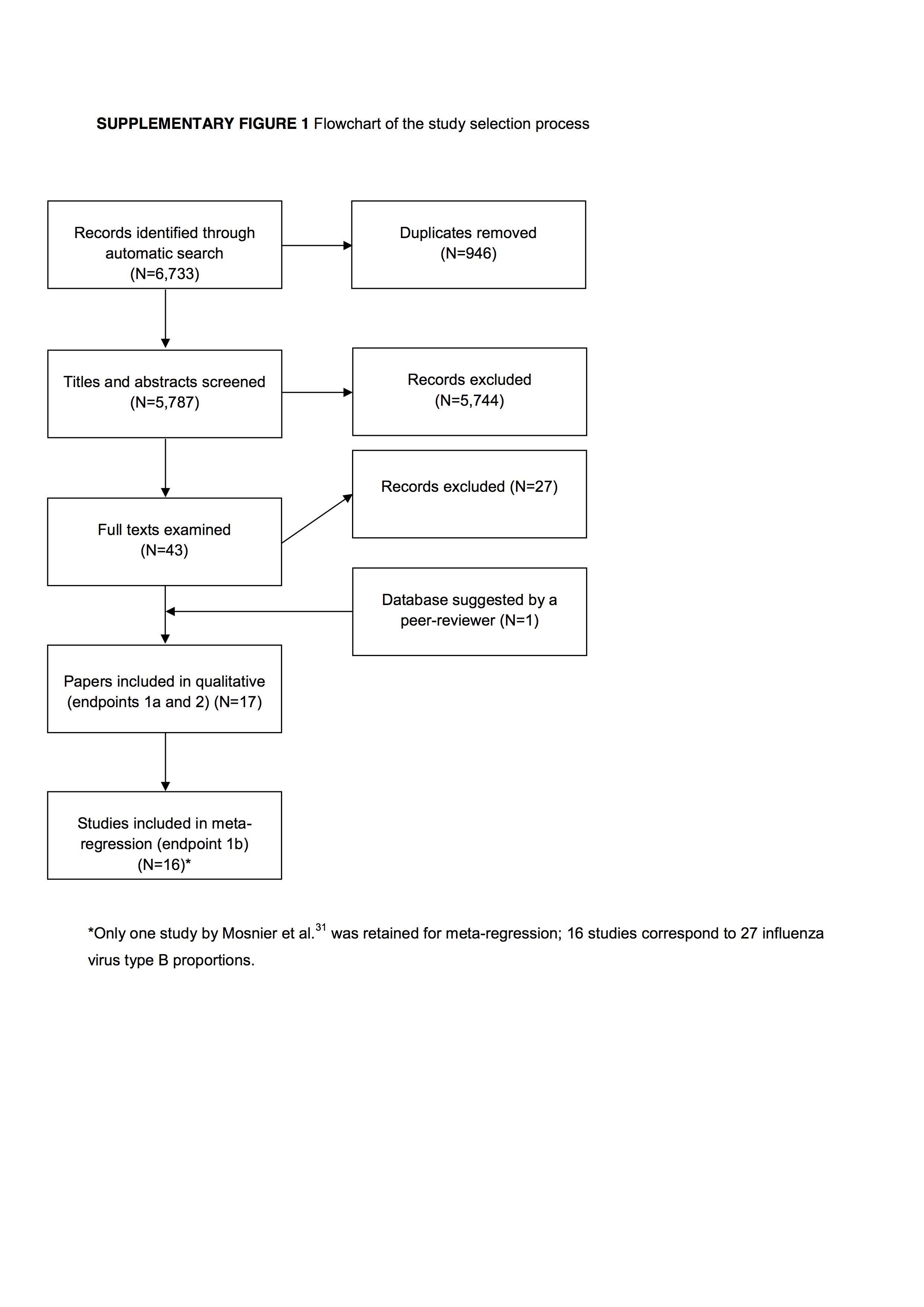

Supplement: Supplementary file 1 [file IRV-12-533-s001.jpg]
