# Supplementary material for: Heterogeneous estimates of influenza virus types A and B in the elderly: Results of a meta‐regression analysis
Source: Influenza Other Respir Viruses. 2018 Mar 23;12(4):533–43. doi: 10.1111/irv.12550 (PMC6005586; doi:10.1111/irv.12550)
Supplement: Supplementary file 2 [file IRV-12-533-s002.docx]

**SUPPLEMENTARY BOX 1** The automatic search strategy

[1] exp Influenzavirus B/

[2] (influenza B or influenza type B or influenza virus B or influenzavirus B or influenza virus type B or influenzavirus type B).tw.

[3] 1 or 2

[4] exp Adult/

[5] Men/

[6] Women/

[7] Retirement/

[8] ((old* or age*) adj3 (people* or person* or adult* or women* or men* or citizen* or residen*)).tw. [9] (pension* or retire* or adult* or aged or elderly or senior* or geriatric*).tw.

[10] long-term care/ or nursing care/ or palliative care/ [11] homes for the aged/ or nursing homes/

[12] nursing home*.tw.

[13] or/4-12

[14] 3 and 13

[15] limit 14 to yr="1990-Current"

[16] remove duplicates from 15
